# Gender differences in caregiver attitudes and unmet needs for activities of daily living (ADL) assistance among older adults with disabilities

**DOI:** 10.1186/s12877-023-04383-2

**Published:** 2023-10-18

**Authors:** Selin Woo, Ying Cui, Suyeon Kim, Mankyu Choi

**Affiliations:** 1 Department of Public Health Science, Graduate School and Transdisciplinary Major in Learning Health Systems, Graduate School, Korea University, 145, Anam-ro, Seongbuk-gu, Seoul, South Korea; 2 School of Health Policy & Management, College of Public Health Science and Transdisciplinary Major in Learning Health Systems, Graduate School, Korea University, 145, Anam-ro, Seongbuk-gu, Seoul, South Korea

**Keywords:** Caregivers, Unmet needs for ADL assistance, Activities of daily living, Gender difference, Older adults

## Abstract

**Background:**

With a rapidly ageing population and a decline in the availability of family caregivers, the number of older adults with disabilities who have unmet long-term care needs is gradually increasing worldwide. However, whether there are gender differences in the association between primary caregivers or their attitudes and unmet needs for activities of daily living (ADL) assistance remains largely unknown.

**Methods:**

This study used the latest 2018 wave of the Chinese Longitudinal Healthy Longevity Survey (CLHLS), containing the data of 1187 older adults with disabilities aged 65 and older, to identify gender differences in the attitudes of primary caregivers toward the unmet needs for ADL assistance among with disabilities adults in China. Binary logistic regression analysis was conducted to determine the effects of primary caregivers and their caregiving attitudes on the unmet care experiences of older adults with ADLs. In addition, a gender-stratified analysis was conducted to compare the differences based on older adults’ gender.

**Results:**

The results revealed that the lack of positive attitudes from primary caregivers might create a situation of unmet needs for ADL assistance among older adults. When family members carry the main burden of care, older adults with disabilities, especially older women, have a lower level of unmet needs for ADL assistance. Therefore, it is important to consider gender-specific interventions to improve ADL assistance among older adults.

**Conclusions:**

The findings suggest that the presence of a family member as a caregiver has a significant effect on unmet needs for ADL assistance in women, highlighting the importance of developing an emotional bond with the caregiver. Given that the availability of informal caregivers, such as family members, is declining, it is crucial to provide financial assistance and formal services, such as paid home services and community-based care services, and reduce the burden on family caregivers to address the unmet needs for ADL assistance among older adults with disabilities in China.

## Background

Population ageing has become a global challenge in developing and developed countries [1]. The number of older adults with disabilities is increasing with the acceleration of ageing [2]. Disability is a generic term for impairments, participation restrictions, and activity limitations [3]. Previous studies have revealed that multiple common diseases [4] and multimorbidities are often observed in older adults and are related to disability [5]. The latest data published by the WHO indicate that more than 100 million older adults over 65 years old will have disabilities by 2030. Disabilities commonly cause several harmful effects, such as reduced physical and mental health, increased accident risk, and decreased quality of life [6].

The most common method for screening disabilities in older adults is to examine their activities of daily living [7]. An unmet situation arises when sufficient assistance is not available. An unmet long-term care need is defined as ‘the difference between the amount of long-term care a person needs and the resources he or she has available to satisfy the need’ [8]. Unmet needs occur in the absence of adequate assistance. In this study, “unmet needs for assistance with activities of daily living” (ADLs) refers to the circumstance in which older adults with disabilities experience limitations in performing ADLs, such as bathing, dressing, toileting, transferring, continence, and feeding, and yet are unable to access the level of assistance they require. This definition aligns with previous research [9–14]. In China, due to ‘China’s One Child Policy‘ adopted in 1979 [15] and the lack of a social nursing insurance system [16] and nursing homes [17], caregivers experience a much heavier burden than before. It has also been reported that older adults living in the community have a higher percentage of unmet ADL needs—approximately 56% in a population-representative study conducted in China [18]. Thus, older adults with disabilities cannot receive sufficient care to support their everyday lives.

Furthermore, unmet ADL needs can vary widely [19] and are associated with multiple factors, including gender [18]. Therefore, this study focuses on exploring the factors influencing unmet needs for assistance with ADL among older adults with disabilities, with particular emphasis on gender differences. Gender differences play a pivotal role in this issue due to their involvement with various factors. Firstly, biological gender differences lead to substantial disparities in health and functionality between women and men [20]. Women are more susceptible to limitations in ADL assistance due to physiological differences, such as menstruation and childbirth, as well as disparities in socioeconomic status and access to healthcare services [20–22]. In previous research [20–22], women exhibit worse health conditions and functional limitations than men, with older women being almost 50% more likely to experience disabilities in basic daily activities and twice as likely to have disabilities in instrumental ADL, compared to older men. Furthermore, their likelihood of limitations in mobility is nearly three times higher than that of older men. Therefore, we will examine the factors associated with unmet needs for assistance with ADL among older adults with disabilities, focusing on gender differences. And Several studies have examined the relationship between caregiver burden and patient characteristics. Numerous characteristics are important for caregivers, including the physical and mental health status of the patient. Furthermore, the level of care burden that partners perceive to be borne by their partners will likely be affected by their perception of the level of support provided by professional care services. To the best of our knowledge, the characteristics of caregivers, including primary caregivers, from the perspective of older adults with ADL limitations, as well as their caregiving attitudes, have not been described in terms of their relative contributions to unmet ADL assistance needs. Although the attitudes of caregivers are expected to improve over time, few studies have examined the effects of gender differences in the association between primary caregivers and their attitudes and unmet needs for ADL assistance.

This study examined the association between primary caregivers and their attitudes and unmet needs for ADL assistance in China. In addition, gender differences in these associations were investigated.

### Literature review

#### Primary caregiver and caregivers’ attitudes for older adults with disabilities

With the increase in the population of older people, there is also a gradual increase in the number of older people with disabilities. As a result, the demand for relevant caregivers is also increasing. However, the number of relevant caregivers is insufficient [23, 24]. Caregivers, mostly family members, are expected to provide caregiving support, medication management in daily life, and financial and psychological support [25].

Presently, relevant research on caregivers focuses primarily on their care status. Previous research has demonstrated that excessive work leads to high stress, physical fatigue, sleep disorders, and social isolation of caregivers [26, 27]. In addition, those who provide care for older adults are more likely to have mental health problems, such as anxiety and depression [26]. Therefore, caregivers must have sufficient resting time to improve the quality of care. Furthermore, digital support services are also necessary for caregivers. A digital support service is an online education platform that provides online education and caring support for caregivers through websites and mobile applications. According to previous studies, online caring support can improve the quality of life of caregivers and older adults [28]. In addition, several studies have shown that caregivers of older adults are more likely to have health problems. Short-term respite care and digital educational support can improve the quality of care provided by caregivers. However, there is insufficient research on the factors that may impact the need for ADL assistance among older adults with disabilities.

#### Unmet ADL assistance needs among older adults with disabilities

ADLs form the foundation of maintaining independence on a daily basis, which includes eating, dressing, bathing, getting in/out of bed, indoor transferring, and toileting [29]. When older adults find it difficult to perform any of these actions independently, it is considered a disability [29]. Unmet ADL assistance needs are defined as a shortage of resources to meet an explicit request for formal or informal support to perform ADLs or instrumental ADLs [30]. For older adults with disabilities who need long-term care, the situation is defined as an unmet need for ADL assistance [31]. The term “unmet ADL assistance needs” implies a gap or discrepancy between the required level of support for ADLs and the actual assistance available. It underscores the notion that despite acknowledging their need for help, these older adults face challenges in obtaining the appropriate level of assistance [9–14]. This concept has significant implications for understanding the barriers faced by older adults with ADL impairments in accessing necessary support services and highlights the importance of addressing these gaps to improve the overall well-being and quality of life of this population.

Presently, relevant research on older adults with disabilities has mainly focused on the use of medical services. In general, research has reported that older adults with disabilities who have an unsatisfied need for assistance have higher outpatient and inpatient times [32]. In addition, the rehospitalization rate of older people with unsatisfactory care experience is about 1.66 times higher than that of older people without unsatisfactory care experience [31]. According to a study, the death rate of older adults with disabilities with unsatisfied assistance experience increased by approximately 10% compared to those with a satisfied assistance experience [33].

However, few studies have investigated the influencing factors of older adults with disabilities who have unmet needs for ADL assistance. Among them, Chen et al. (2021) [29] found that family care resources, household income, loneliness, and the number of ADLs affected the unmet needs for ADL assistance of older people with disabilities. In addition, economic status, having someone other than a family member as the primary caregiver, caring attitude of caregivers, timely medication, self-rated health, and self-rated life satisfaction impact unmet needs for ADL assistance [33]. In addition, when caregivers maintain a positive attitude, the likelihood of older individuals with disabilities encountering unmet needs diminishes by 78% in rural regions and 77% in urban areas.

#### Andersen’s expanded behavioral model of health service use

Andersen’s expanded behavioral model of health service use is an extension of the framework originally proposed by Andersen and Newman in 1995 [34] and has undergone various revisions over time [35]. We utilized the sixth revision [36], a multilevel model incorporating individual and environmental determinants of health service utilization, which are determined by three factors: predisposing, enabling, and need [37].

Predisposing factors can be divided into those at the individual and contextual levels. Individual predisposing factors include demographic factors (e.g., age and sex/gender), social factors (e.g., education, occupation, ethnicity, and social relationships), and mental factors in terms of health beliefs (e.g., attitudes, values, and knowledge related to health and related services). Contextual predisposing factors include the demographic and social composition of communities (e.g., educational levels and crime rates), as well as collective and organizational values, cultural norms, and political perspectives. In addition, enabling factors encompass financial and organizational aspects that facilitate access to medical services. Individual financing factors involve income and wealth, which determine the ability to afford healthcare services. Organizational factors pertain to whether individuals have a consistent healthcare provider and the characteristics of that provider. At a contextual level, financing encompasses the resources allocated for health services within the community, as well as the prevailing health policies. Organization pertains to the quantity, types, locations, structures, and distribution of health services and personnel. Health policies also fall under the category of contextual enabling factors. Finally, need factors refer to the factors that make people feel the necessity of using medical services. Regarding individual need factors, two distinct aspects are considered: the perceived need for health services, which refers to how individuals perceive and experience their overall health status, and the assessed need, which involves professional assessment and objective measurements of patients. Regarding contextual need factors, the model distinguishes between environmental demand characteristics, which encompass factors such as occupational injuries, transportation-related injuries, and mortality due to crime, and population health indicators. Population health indicators include epidemiological measures such as mortality rates, morbidity rates, and disability rates [36].

Zhu (2015) [18] utilized Andersen’s behavioral model of health service use to investigate the predisposing, enabling, and need factors contributing to unmet needs associated with ADL disabilities in long-term care. The findings revealed significant aspects of unmet needs associated with ADL disabilities. Among the enabling factors, economic status, having a non-family member as the primary caregiver, caregivers’ willingness to provide care, and timely medication were significant factors. Among the need factors, self-rated health and self-rated life satisfaction were significant factors. Furthermore, the study highlighted the existence of urban–rural disparities. For urban residents, significant factors included age (predisposing), primary caregiver (enabling), ADL disabilities, expectation of access to community-based care services, and optimism (need). Conversely, for rural residents, the significant factors were gender (predisposing) and cognitive function (enabling).

Andersen’s expanded behavioral model of health service use is frequently employed in various aspects of healthcare utilization research, such as medical use [38], unmet healthcare needs [39], community care utilization [18], and home care services [40]. Most studies using this model are about medical use, as opposed to the factors that affect unsatisfactory care experience, as examined in this study. In existing pilot studies, Andersen’s expanded behavioral model of health service use is applied to classify the impact of predisposing, enabling, and need factors on medical use. The review of relevant pilot studies using the dependent variables is as follows: First, a representative example is a study using Andersen’s expanded behavioral model of health service use to observe the impact of inpatient and outpatient medical services use. According to previous research [41], age, as a predisposing factor, affects inpatient medical expenses, outpatient medical expenses, and nursing costs. All factors showed remarkable results in a study that distinguished outpatient and inpatient medical use for observation [42]. Among the predisposing factors affecting inpatient care use, gender, age, and marital status were more significant. Among the enabling factors, education level, economic level, and insurance type were most important. In addition, among the need factors, chronic disease and disability status were more significant.

According to Mohaqeqi Kamal (2022) [38], predisposing and enabling factors affected the utilization rate of outpatient care. Among the enabling factors, monthly income and living status had an impact. Compared with the group with a monthly income of less than $100, the group earning between $100 and $200, and the group earning more than $200, the average monthly outpatient medical utilization rate was higher. Older adults living with a partner had a 2.36 times higher utilization rate on the outpatient health services than those who lived alone [29]. Among the predisposing factors, the utilization rate of outpatient medical care for people with a high school education or above is 2.65 times that of the illiterate [38].

The research objective of the relevant literature is based on the use of family convalescent services with older people. According to research [40], among the enabling factors, economic factors significantly impacted the use of family health services for older adults. Specifically, among the economic factors, the impact on the utilization rate of family medical services varies according to whether the main source of income is the pension, whether there is an economic surplus, and the main consumption type [40]. Those who live mainly on a pension, have inadequate economic conditions and have water, electricity, transportation, and medical-related expenses as their main consumption types have a high probability of using family medical services [40]. Furthermore, according to Willis and Glaser (2013) [43], race of predisposing factors significantly impacts the choice of family convalescence. Compared to the White British group, the Indian group had significantly more family care.

Some studies have also discussed the influence of Andersen’s expanded behavioral model of health service use on unsatisfactory medical use. According to Pan et al. (2022) [44], the predisposing factors of age, marital status, place of domicile, and subjective health status, as well as the need factor of chronic disease presence, significantly affect unsatisfactory medical utilization. Specifically, according to Pan et al. (2022) [44], adults under 60 years old, unmarried, living in rural areas, with better subjective health, and no chronic diseases are more likely to choose self-treatment instead of medical treatment. The better the subjective health, the less chronic the disease and the lower the utilization rate of hospitalization. On the contrary, compared with other medical insurance, such as hospitalization services, the utilization rate of inpatient medical care is 1.457 times higher with Urban Employee Basic Medical Insurance [44].

Therefore, we utilize primary caregivers and their attitudes as the key independent variable, predisposing, enabling, and need factors as control variables to examine the gender difference in unmet need for ADL assistance among older adults with disabilities.

#### Present study

It remains uncertain whether the primary caregivers and their attitudes of primary caregivers towards older adults with disabilities are associated with the unmet needs for ADL assistance among older adults with disabilities. In particular, we know very little about the gender patterns of these relationships.

Therefore, this study examined the independent associations between primary caregivers and their attitudes and the unmet needs for ADL assistance among older adults with disabilities to identify whether the associations were gender-specific. The independent variables in this study include primary caregivers and their attitudes, and the control variables include predisposing, enabling, and need factors. The predisposing factors examined were age, gender, marital status, and educational level. The enabling factors included current residence and financial independence. Need factors included self-perceived health status, the number of ADL impairments, and having one or more chronic diseases.

**Fig. 1:**
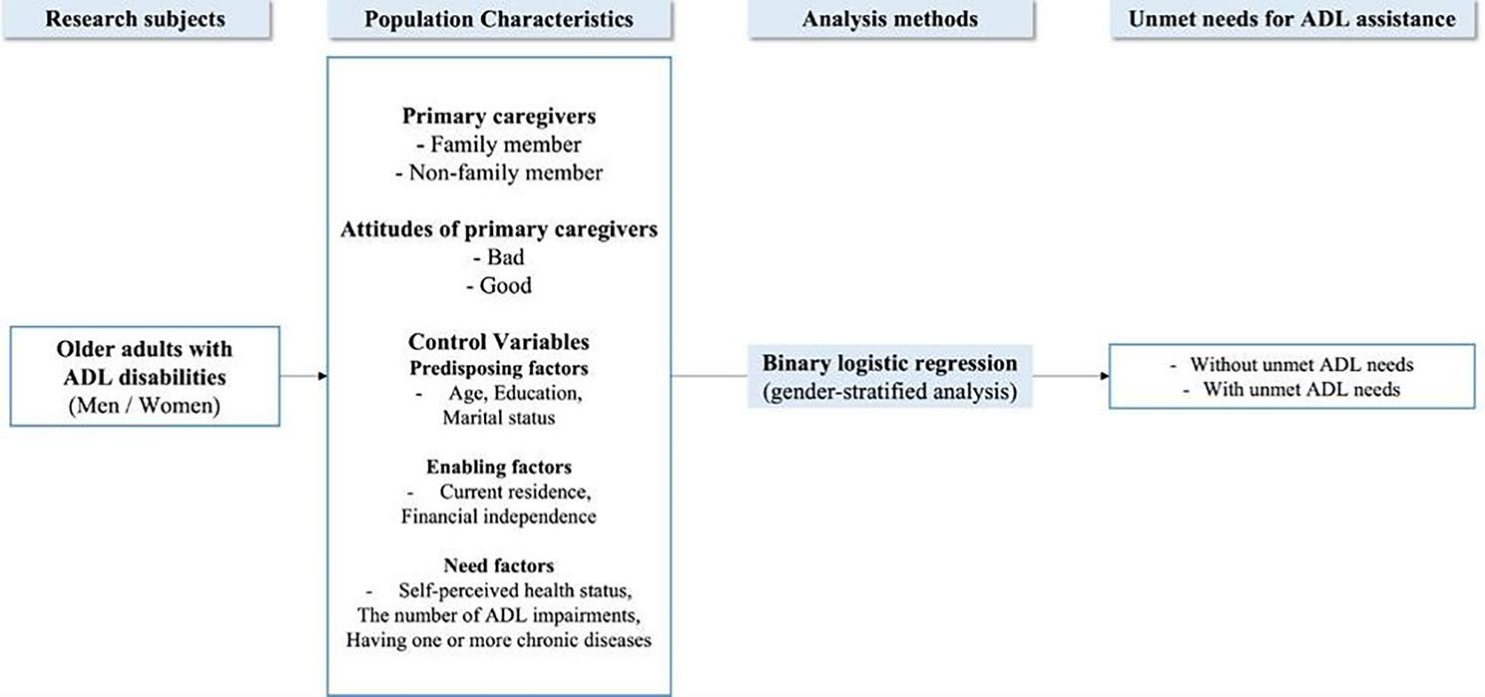
Conceptual model

The first hypothesis of this study is that primary caregivers and their attitudes affect the unmet needs for ADL assistance among older adults with disabilities. In addition, there are fewer unmet needs for ADL assistance when primary caregivers are mainly family members and when primary caregivers have better attitudes. The second hypothesis is that the impact of primary caregivers and their attitudes on the unmet needs for ADL assistance is gender-specific. The study protocol is illustrated in Fig. 1.

## Methods

### Data collection

The data used in this study were obtained from the Chinese Longitudinal Healthy Longevity Survey (CLHLS) conducted in 2018. The CLHLS, established in 1998, is conducted every three years. The 8th wave of the CLHLS—2018—includes several counties/cities in 22 of the 31 provinces and Chenmai County in Hainan Province, which was included in the CLHLS as the accuracy of age reporting was similar to that in the other 22 provinces [45].

The CLHLS is designed to investigate the influence of social, behavioral, environmental, and genetic factors, as well as their interactions, on healthy longevity [46]. The 8th wave of the CLHLS was carried out by Peking University in collaboration with the Centers for Disease Control and Prevention [47].

The CLHLS employed a multi-stage targeted disproportionate random sampling method. Data collection involved conducting face-to-face interviews and administering basic physical ability tests in the respondents’ homes [48]. The survey encompassed several key components, including basic information about the older adult population, assessing initial health status, evaluating cognitive ability, examining lifestyle factors, assessing the ability to perform ADL, and obtaining self-assessment reports from the participants [46].

Remarkably, the refusal rate for interviews among older adults in China was exceptionally low, with only approximately 2% of those individuals who were not excessively frail owing to illness and required proxy assistance choosing to decline participation. To enhance data quality, rigorous procedures were implemented, including data cleaning and evaluation, validity and reliability testing, as well as the analysis of non-response and missed interviews for follow-up surveys. As a result, the collected data demonstrated reliability and satisfactory quality [46].

When selecting the cities for the study, certain criteria were considered, one of which was the importance of reliable and accurate age reporting by the population residing in those cities. As age was a crucial variable in the data analysis, it was essential to ensure that the age information obtained on-site was trustworthy. Consequently, certain areas were excluded from the survey owing to significant misreporting of age in the census data [47].

Adults above 65 years old who required partial or full assistance with at least one of the six ADLs - bathing, dressing, indoor transfer, toilet, eating and continence - were selected for the study. A total of 1,187 patients were enrolled in the study. Moreover, individuals with cognitive disabilities were not excluded; proxy respondents were utilized.

### Unmet needs for ADL assistance as the dependent variable

In this study, older adults with disabilities were classified as having unmet needs for ADL assistance if they reported not receiving the necessary assistance, expressing a need for additional help, or experiencing delays in task completion owing to a lack of sufficient support. In the CLHLS survey, patients were asked the following question: ‘Does the assistance provided by caregivers meet your needs?’ The replies had three levels: (1) not met, (2) partially met, and (3) fully met. Next, answers (1) and (2) were combined and classified as ‘with unmet ADL needs,’ and (3) were classified as ‘without unmet ADL needs.’

### Primary caregivers and their attitudes as the key Independent variable

The patients were asked: ‘Who is the primary caregiver when you need assistance in bathing, dressing, toileting, indoor transferring, continence, and eating?’ The participants were then asked how they knew their primary caregivers. The responses included: (1) spouse, (2) son, (3) daughter-in-law, (4) daughter, (5) son-in-law, (6) unmarried son or daughter, (7) grandchild(ren), (8) relative(s), (9) friend(s) or neighbour(s), (10) social services, (11) housekeeper, and (12) nobody (excluded from the study). Subsequently, responses with (1–8) were classified as family members and (9–11) as non-family members.

The attitudes of the primary caregivers were measured based on the following question: ‘What is your primary caregiver’s attitude when she/he takes care of you?’ Responses to this question were classified into five categories: (1) willing, (2) impatient, (3) need respite care, (4) unwilling, and (5) do not know (excluded from the study). Answers (2–4) were combined and identified as ‘bad’, and (1) was identified as ‘good’.

### Control variables

Based on Andersen’s expanded behavioral model of health service use, control variables were divided into predisposing, enabling, and need factors. Predisposing factors included sociodemographic variables such as age, marital status, and education. Marital status was classified as unmarried, married, or single, with single including divorced or widowed. Education was measured as 0–6 years, 7–9 years, 10–12 years, and > 13 years. enabling factors include current residence and financial independence. According to the urban-rural classification method defined by the National Bureau of Statistics [45, 49], current residence was classified as rural or urban [50, 51]. Financial independence was measured as reliance on others and independence. Need factors include self-perceived health status, the number of ADL impairments and having one or more chronic diseases. The question, ‘How do you rate your health at present?’ was asked to measure self-perceived health status. The responses were measured as ‘very poor’, ‘poor’, ‘general’, ‘good’, and ‘very good’. Higher scores indicated better self-rated health and life satisfaction. The number of ADL impairments was determined by counting the number of ADLs that older adults with disabilities could not perform. The number of having one or more chronic diseases was determined by counting the number of chronic diseases of older adults with disabilities.

### Data analysis

Data analyses were performed using SPSS 18.0. Frequency was used to describe disability status, sociodemographic variables, and other variables. Binary logistic regression analysis was used to identify the significant factors associated with unmet needs for ADL assistance.

In model 1, primary caregivers and their attitudes were considered independent variables. In model 2, primary caregivers and their attitudes were considered independent variables, and predisposing factors (gender, age, education, and marital status) were considered control variables. In model 3, primary caregivers and their attitudes were considered independent variables, and predisposing and enabling factors (current residence and financial independence) were considered control variables. Finally, in model 4, primary caregivers and their attitudes were considered independent variables, and predisposing, enabling, and need factors (self-perceived health status, number of ADL impairments, and having one or more chronic diseases) were considered control variables.

Analyses were conducted on the total sample and stratified by gender. Statistical significance was set at *p* < 0.05, based on two-tailed tests. Multicollinearity between the independent variables was assessed using variance inflation factors (VIF; reference value of 10) before interpreting the final output.

## Results

Table 1 shows the characteristics of the participants. Of the 1187 participants in the study, 542 (45.7%) had unmet ADL needs, and 645 (54.3%) had no unmet ADL needs. The unmet need for ADL assistance was 45.3% (185) for older men and 45.8% (357) for older women with disabilities. A total of 1039 (87.5%) primary caregivers for older adults with disabilities were family members, and 148 (12.5%) were non-family members. Eighty-six (7.2%) participants experienced a negative attitude, and 1101 (92.8%) experienced a positive attitude. A total of 408 (34.4%) were men, and 779 (65.6%) were women. Regarding education, 1044 (88%) patients had received education for less than six years, 69 (5.8%) for 7–9 years, and 41 (3.5%) for 10–12 years. Further, 83.3% were single (divorced or widowed), 16.4% were married, and 0.3% were unmarried. In addition, 66.5% of the participants were lived in rural areas. Among the participants, 33.5% lived in a urban. And 85.9% needed economic self-support. Nearly 40% of the respondents needed assistance with one daily life activity, nearly 13% with two activities, 9% with three activities, 13% with four activities, and more than 25% with more than five activities. Nearly 70% of the respondents having one or more chronic diseases.

**Table 1:** *Characteristics of the participants*

Variables	Total		Men		Women		P
	n	%	n	%	n	%	
Unmet needs for ADL assistance							0.874
Without unmet ADL needs	645	54.3%	223	54.7%	422	54.2%	
With unmet ADL needs	542	45.7%	185	45.3%	357	45.8%	
Primary caregivers							0.257
Family member	1039	87.5%	351	86%	688	88.3%	
Non-family member	148	12.5%	57	14%	91	11.7%	
Attitudes of primary caregivers							0.918
Bad	86	7.2%	30	7.4%	56	7.2%	
Good	1101	92.8%	378	92.6%	723	92.8%	
*predisposing factors*							
Age							0.222
	86.09	(SM = 11.492)	86.66	(SM = 11.639)	85.80	(SM = 11.411)	
Education							< 0.001
0–6 years	1044	88%	313	76.7%	731	93.8%	
7–9 years	69	5.8%	42	10.3%	27	3.5%	
10–12 years	41	3.5%	30	7.4%	11	1.4%	
More than 13 years	33	2.5%	23	5.6%	10	1.3%	
Marital status							< 0.001
Unmarried	3	0.3%	3	0.7%	0	0.0%	
Married	195	16.4%	121	29.7%	74	9.5%	
Single	989	83.3%	284	69.6%	705	90.5%	
*enabling factors*							
Current residence							< 0.001
Urban area	398	33.5%	163	40.0%	235	30.2%	
Rural area	789	66.5%	245	60.0%	544	69.8%	
Financial independence							**0.806**
Independent	1020	85.9%	352	86.3%	668	85.8%	
Dependent	167	14.1%	56	13.7%	111	14.2%	
*need factors*							
Self-perceived health status							0.935
Very good	108	9.1%	38	9.3%	70	9.0%	
Good	357	30.1%	123	30.1%	234	30.0%	
General	435	36.6%	147	36.0%	288	37.0%	
Poor	225	21.5%	87	21.3%	168	21.6%	
Very poor	32	2.7%	13	3.2%	19	2.4%	
The number of ADL impairments							0.036
1	472	39.8%	163	40.0%	309	39.7%	
2	154	13.0%	68	16.7%	86	11.0%	
3	108	9.1%	37	9.1%	71	9.1%	
4	155	13.1%	56	13.7%	99	12.7%	
5	178	15.0%	53	13.9%	125	16.0%	
6	120	10.1%	31	7.6%	89	11.4%	
Having one or more chronic diseases							0.023
No	363	30.6%	108	26.5%	255	32.7%	
Yes	824	69.4%	300	73.5%	524	67.3	

*Notes*: ADL: activities of daily living

Further, 86% (351) of primary caregivers for men older adults with disabilities were family members, and 14% (57) were non-family members. In addition, 92.6% (378) reported that the caregivers had a good caregiving attitude, and 7.4% (30) reported that they were in poor care. Only 11.7% (91) of older women with disabilities were cared for primarily by someone outside the family, and 88.3% (668) were cared for by family members. Of the participants, 7.2% (56) experienced a good attitude, and 92.8% (723) experienced a bad attitude.

As seen in Table 1, older women, compared to older men, were more likely to receive less than six years of education, be single, live in rural areas, have greater ADL impairments and no more than one chronic disease. However, there were no significant gender differences in unmet needs for ADL assistance, primary caregivers, attitudes of primary caregivers, age, or financial independence.

< Insert Table 1 about here >.

Table 2 shows the factors that affect the unmet needs for ADL assistance. This study used the VIF to identify multicollinearity in the regression model, and the results showed that all VIFs were less than 1.3. Therefore, there was no multicollinearity among the variables. In models 1–4, better attitudes from primary caregivers were positively associated with met ADL assistance needs. However, there was no significant association between primary caregivers and unmet needs for ADL assistance in models 1, 2, and 4. However, model 3 showed that non-family members acting as caregivers (OR = 1.603; 95% CI = 1.081–2.377) were positively associated with unmet needs for ADL assistance. The results from model 1 were consistent with the above findings (attitudes of primary caregivers: OR = 0.132; 95% CI = 0.072–0.240).

**Table 2:** *Regression results for unmet needs for ADL assistance among the whole sample group*

Variables	Model 1		Model 2		Model 3		Model 4	
	Exp(β)(OR)	95% CI	Exp(β)(OR)	95% CI	Exp(β)(OR)	95% CI	Exp(β)(OR)	95%CI
Primary caregivers								
**Family member**								
**Non-family member**	1.061	0.747–1.507	1.247	0.857–1.814	1.603*	1.081–2.377	1.444	0.958–2.178
**Attitudes of primary caregivers**								
**Bad**								
**Good**	0.132 ***	0.072–0.240	0.132***	0.072–0.242	0.148***	0.080–0.274	0.148***	0.079–0.279
***predisposing factors***								
**Age**								
			0.998	0.988–1.009	0.998	0.988–1.009	0.996	0.986–1.007
**Education**								
**0–6 years**								
**7–9 years**			0.663	0.393–1.118	0.880	0.512–1.511	0.893	0.511–1.559
**10–12 years**			0.551	0.277–1.093	0.726	0.361–1.462	0.735	0.358–1.509
**More than 13 years**			0.457	0.207–1.009	0.605	0.272–1.347	0.562	0.246–1.282
**Marital status**								
**Unmarried**								
**Married**			1.250	0.069–23.617	1.796	0.098–33.012	1.466	0.083–25.926
**Single**			1.161	0.065–20.806	1.691	0.093–33.782	1.285	0.073–22.536
***enabling factors***								
**Current residence**								
**Urban area**								
**Rural area**					1.927***	1.458–2.547	1.708***	1.270–2.297
**Financial independence**								
**Independent**								
**Dependent**					1.828***	1.285-2.600	1.559*	1.073–2.265
***need factors***								
**Self-perceived health status**								
**Very good**								
**Good**							1.273	0.773–2.098
**General**							1.898*	1.162–3.099
**Poor**							2.366*	1.392–4.023
**Very poor**							3.226*	1.254–8.298
**The number of ADL impairments**								
							1.245***	1.158–1.338
**Having one or more chronic diseases**								
**No**								
**Yes**							0.620***	0.469–0.819

*Notes*: ADL: activities of daily living. OR: odds ratio

*Significance levels*: *P < 0.05, **P < 0.01, ***P < 0.001

In model 2, good attitudes from caregivers (OR = 0.132; 95% CI = 0.072–0.242) were negatively associated with unmet needs for ADL assistance.

In model 3, non-family member primary caregivers (OR = 1.603; 95% CI = 1.081–2.377), living in rural areas (OR = 1.927; 95% CI = 1.458–2.547), and economic dependence (OR = 1.828; 95% CI = 1.285–2.600) were positively associated with unmet needs for ADL assistance. In contrast, better attitudes from primary caregivers (OR = 0.148; 95% CI = 0.080–0.274) were negatively associated with unmet needs for ADL assistance.

In model 4, living in rural areas (OR = 1.708; 95% CI = 1.270–2.297), economic dependence (OR = 1.559; 95% CI = 1.075–2.265), general self-perceived health status (OR = 1.898; 95% CI = 1.162–3.099), poor self-perceived health status (OR = 2.366; 95% CI = 1.392–4.023), very poor self-perceived health status (OR = 3.226; 95% CI = 1.245–8.298), and a higher number of ADL impairments (OR = 1.245; 95% CI = 1.158–1.338) were positively associated with unmet needs for ADL assistance. Conversely, better attitudes from primary caregivers (OR = 0.144; 95% CI = 0.958–2.178) and having one or more chronic diseases (OR = 0.620; 95% CI = 0.469–0.819) were negatively associated with unmet needs for ADL assistance.

< Insert Table 2 about here >.

Tables 3 and 4 show the gender difference test for associations between unmet needs of ADL assistance and influencing factors among older adults with disabilities. Table 3 shows the regression results stratified by gender for unmet needs for ADL assistance among older men. For older men with disabilities, models 1–4 demonstrated that better attitudes from primary caregivers were negatively related to unmet needs for ADL assistance. However, there was no significant association between primary caregivers and unmet needs for ADL assistance among older men in models 1–4.

**Table 3:** *Regression results stratified by gender for unmet needs for ADL assistance among older men*

Variables	Men										
	Model 1		Model 2		Model 3				Model 4		
	Exp(β) (OR)	95% CI	Exp(β) (OR)	95% CI	Exp(β) (OR)			95% CI	Exp(β) (OR)		95% CI
**Primary caregivers**											
**Family member**											
**Non-family member**	0.779	0.428–1.417	0.894	0.469–1.703	1.14		0.574–2.252		1.009	0.497–2.048	
**Attitudes of primary caregivers**											
**Bad**											
**Good**	0.024***	0.003–0.179	0.021***	0.003–0.171	0.024***		0.003–0.195		0.023***	0.003–0.191	
**predisposing factors**											
**Age**											
			0.994	0.976–1.011	0.99		0.975–1.011		0.992	0.974–1.011	
**Education**											
**0–6 years**											
**7–9 years**			0.68	0.335–1.379	0.89		0.424–1.846		0.881	0.412–1.885	
**10–12 years**			0.743	0.326–1.693	0.95		0.408–2.205		1.006	0.420–2.413	
**More than 13 years**			0.509	0.187–1.388	0.65		0.237-1.800		0.574	0.201–1.641	
**Marital status**											
**Unmarried**											
**Married**			2.8	0.056-140.666	3.9	0.069-220.929			3.851	0.085-175.118	
**Single**			3.146	0.063-157.095	4.39		0.078-246.492		4.555	0.101-205.294	
***enabling factors***											
**Place of residence**											
**Urban area**											
**Rural area**					1.665*		1.029–2.692		1.736*	1.043–2.889	
**Financial independence**											
**Independent**											
**Dependent**					2.653**		1.371–5.132		2.239*	1.122–4.468	
***need factors***											
**Self-perceived health status**											
**Very good**											
**Good**									1.244	0.554–2.791	
**General**									1.151	0.521–2.541	
**Poor**									2.396*	1.003–5.724	
**Very poor**									2.834	0.579–13.863	
**The number of ADL impairments**											
									1.217*	1.063–1.393	
**Having one or more chronic diseases**											
**No**											
**Yes**									0.858	0.514–1.433	

*Notes*: ADL: activities of daily living. OR: odds ratio

*Significance levels*: *P < 0.05, **P < 0.01, ***P < 0.001

Model 1 revealed that a good attitude of caregivers (OR = 0.024; 95% CI = 0.003–0.179) was negatively related to the unmet needs for ADL assistance among older men. However, there was no significant association between primary caregivers (OR = 0.779; 95% CI = 0.428–1.417) and the unmet needs for ADL assistance among older men.

Model 2 revealed that a good attitude of caregivers (OR = 0.021; 95% CI = 0.003–0.171) was negatively related to the unmet needs for ADL assistance among older men. Conversely, there was no significant association between primary caregivers (OR = 0.894; 95% CI = 0.469–1.703) and unmet needs for ADL assistance among older men.

In model 3, living in rural areas (OR = 1.665; 95% CI = 1.029–2.692) and financial dependence (OR = 2.653; 95% CI = 1.371–5.132) were risk factors for older men with unmet needs for ADL assistance. However, a good attitude (OR = 0.024; 95% CI = 0.003–0.195) was a protective factor.

The results from model 4 indicate that living in rural areas (OR = 1.736; 95% CI = 1.043–2.889), economic dependence (OR = 2.239; 95% CI = 1.122–4.468), poor self-perceived health status (OR = 2.396; 95% CI = 1.003–5.724), very poor self-perceived health status (OR = 3.630; 95% CI = 1.095–12.037) and a higher number of ADL impairments (OR = 1.243; 95% CI = 1.139–1.356) were risk factors for older men with unmet needs for ADL assistance. A good attitude of primary caregivers (OR = 0.023; 95% CI = 0.003–0.191) was a protective factor for older men with unmet needs for ADL assistance.

Table 4 shows the unmet needs for ADL assistance among older women. For older women, models 1–4 confirmed that positive attitudes of caregivers indicated fewer unmet needs for ADL assistance. Primary caregivers were not significantly correlated in models 1 and 2. For models 3 and 4, the primary caregivers were family members and good attitudes from caregivers related to the unmet needs for ADL assistance among older women.

In model 1, good attitudes of caregivers (OR = 0.203; 95% CI = 1.062–1.389) indicated less unmet needs for ADL assistance among older women. However, there was no significant association between primary caregivers (OR = 1.241; 95% CI = 0.799–1.929) and unmet needs for ADL assistance among older women.

In model 2, positive attitudes of caregivers (OR = 0.208; 95% CI = 0.108–0.401) indicated fewer unmet needs for ADL assistance among older women. However, primary caregivers and predisposing factors (gender, age, education, and marital status) were not significantly associated with unmet needs for ADL assistance among older women.

In model 3, non-family member caregivers (OR = 1.940; 95% CI = 1.178–3.915), living in rural areas (OR = 2.062; 95% CI = 1.454–2.922), and financial dependence (OR = 1.54; 95% CI = 1.009–2.352) were risk factors for older women with unmet needs for ADL assistance. In addition, a good attitude (OR = 0.235; 95% CI = 0.121–0.457) was a protective factor.

In model 4, non-family member primary caregivers (OR = 1.698; 95% CI = 1.002–2.877), living in rural areas (OR = 1.614; 95% CI = 1.110–2.347), general self-perceived health status (OR = 2.623; 95% CI = 1.386–4.964), poor self-perceived health status (OR = 2.580; 95% CI = 1.301–5.118), and a higher number of ADL impairments (OR = 1.268; 95% CI = 1.154–1.372) were risk factors. A good attitude of primary caregivers (OR = 0.244; 95% CI = 0.123–0.485) was a protective factor for older women with unmet needs for ADL assistance. Conversely, having one or more chronic diseases (OR = 0.620; 95% CI = 0.469–0.819) were negatively associated for older women with unmet needs for ADL assistance.

**Table 4:** *Regression results stratified by gender for unmet needs for ADL assistance among older women*

Variables	Women							
	Model 1		Model 2		Model 3		Model 4	
	Exp(β) (OR)	95% CI	Exp(β) (OR)	95% CI	Exp(β) (OR)	95% CI	Exp(β) (OR)	95% CI
**Primary caregivers**								
**Family member**								
**Non-family member**	1.241	0.799–1.929	1.52	0.942–2.438	1.940**	1.178–3.915	1.698*	1.002–2.877
**Attitudes of primary caregivers**								
**Bad**								
**Good**	0.203***	0.105–0.392	0.208***	0.108–0.401	0.235***	0.121–0.457	0.243***	0.121–0.487
***predisposing factors***								
**Age**								
			1	0.989–1.014	1.002	0.989–1.015	1	0.986–1.013
**Education**								
**0–6 years**								
**7–9 years**			0.62	0.272–1.405	0.836	0.358–1.956	0.798	0.329–1.938
**10–12 years**			0.22	0.047–1.075	0.315	0.065–1.522	0.300	0.059–1.518
**More than 13 years**			0.4	0.098–1.638	0.554	0.132–2.322	0.536	0.119–2.413
**Marital status**								
**Unmarried**								
**Married**								
**Single**			0.82	0.497–1.345	0.863	0.519–1.435	0.724	0.428–1.224
***enabling factors***								
**Place of residence**								
**Urban area**								
**Rural area**					2.062***	1.454–2.922	1.614*	1.110–2.347
**Current residence**								
**Independent**								
**Dependent**					1.54	1.009–2.352	1.316	0.835–2.076
***need factors***								
**Self-perceived health status**								
**Very good**								
**Good**							1.343	0.704–2.564
**General**							2.623*	1.386–4.964
**Poor**							2.580*	1.301–5.118
**Very poor**							3.630*	1.095–12.037
**The number of ADL impairments**								
							1.243***	1.139–1.356
**Having one or more chronic diseases**								
**No**								
**Yea**							0.525***	0.373–0.737

*Notes*: ADL: activities of daily living. OR: odds ratio

*Significance levels*: *P < 0.05, **P < 0.01, ***P < 0.001

## Discussion

This study used CLHLS data, the only data source in China that includes older adults with disabilities who fail to meet their assistance needs, to analyze the influence of the main caregivers and their attitudes on the unmet assistance needs of older adults with disabilities. Furthermore, from the gender perspective, it specifically analyses the gender differences of the main caregivers and their care attitude toward older adults with disabilities.

At present, research on older adults with disabilities is being actively carried out, but research reflecting the care characteristics of caregivers is insufficient. Therefore, in this study, the main caregivers were divided into two categories: family members and personnel other than family members. Furthermore, the attitude of caregivers can be divided into good and bad. The patients were people over 65 years with one or more ADL disorders. Through stratified analysis of different gender, the gender differences of the main caregivers and their attitude towards the unsatisfied assistance experience of older adults with disabilities were clarified. In addition, while analyzing the results, relevant programs that can eliminate the needs of older adults with disabilities should be actively explored.

The results showed that the main caregivers were closely related to their attitudes and the needs of older adults with disabilities for assistance. Moreover, even when confounding factors were controlled, the results remained unchanged.

First, according to the analysis results of the main caregivers, when the main caregivers were family members, the possibility of experiencing unsatisfied assistance for all the older adults with disabilities was significantly reduced compared with that for the main caregivers who were not family members. These findings are consistent with previous research [18]. Research indicates that when care is provided by daughters and sons-in-law, the likelihood of older adults with disabilities encountering unmet needs is reduced compared to when care is provided by non-family members. This result is related to the closeness and comfort of home care. Therefore, when non-family members serve as the main caregivers for older people with disabilities, it is necessary to provide counselling services that can improve the intimacy between caregivers and older adults with disabilities. In this regard, Fu and Chui (2017) [52] explained the same.

Furthermore, this study found that older people prefer to receive convalescence (HCBC) in a familiar environment than in institutions. Meng and Davidson (2021) [53] also revealed that receiving convalescence in a familiar environment positively reduced the unmet aid needs of older adults with disabilities. In addition, Zhang and Hou (2018) [54] pointed out that regular visits and health checkups by professional caregivers in the community to older people at home can improve intimacy with them and positively impact their health. This study confirmed a gender difference between the main caregivers and their care attitude towards older adults with disabilities who do not meet their assistance needs. Therefore, according to the gender classification, the main caregivers only produced statistically significant results for women-older adults with disabilities. This result is consistent with previous research. Compared with older men, older women with disabilities have lower financial ability and health levels and are more sensitive to emotions, depending more on family care [55]. Furthermore, research findings [20–22] indicate that women tend to be more susceptible to age-related physical health issues compared to men. This suggests that women, who are relatively more vulnerable to physical health issues, may encounter difficulties in performing specific ADL tasks such as bathing, dressing, and eating. Therefore, it is necessary to develop a care system that can improve the intimacy between caregivers and older adults with disabilities, considering gender differences. Women exhibit susceptibility to physical health issues and are more affected by emotional issues compared to men. Given that family members often assume the role of primary caregivers for women, establishing an emotional bond with caregivers is considered crucial. Furthermore, it is necessary to develop physical therapy programs that specifically target activities such as dressing, bathing, and using the bathroom, as these interventions can significantly contribute to women’s independence and overall quality of life.

Second, from the research results of the attitudes of caregivers, the group that experienced poor care attitude was more likely to experience unsatisfactory assistance than the group that experienced good care attitude. This finding is consistent with Zhu (2015) [18], that is, the caring attitude of caregivers will affect the care experience of older adults with disabilities. Therefore, it is necessary to provide professional education for caregivers and formulate relevant programs to alleviate the burden of care pressure for older adults with disabilities to improve the situation.

Specifically, it is important to provide relevant educational support. Relevant literature shows that when caregivers are taught professional and emergency treatment knowledge, their care pressure is reduced. Research Zhong and Nicholas (2020) [56] showed that the stress value of caregivers was low in the group with professional training. In addition, research has shown that online professional education implemented through the network positively eliminates the negative emotions of caregivers. Considering the current COVID-19 situation in China, building an online knowledge education platform is necessary.

In addition, nationwide long-term care insurance is needed. As the population ages, governments must do more to ensure the health and well-being of older adults. One of them is long-term care insurance. Long-term care insurance plays an important role in the welfare of older adults with disabilities in ADLs by providing much needed support in the form of in-home care and rehabilitation services. This support has a positive impact on not only ADLs but also the attitudes of their primary caregivers. Furthermore, a study [57] found that long-term care insurance, as the main public sector care system, can adequately provide the necessary in-home services. Therefore, the nationwide expansion of long-term care insurance home services for older adults will help alleviate the burden of dependency and improve welfare levels in China. Although there have been trials of long-term convalescent insurance in some areas, there has not been a nationwide long-term care system such as the long-term convalescent insurance in Korea and nursing insurance in Japan. In South Korea, with the implementation of the long-term care insurance system, the burden of family care and regional inequalities in the availability of care services for older people has been reduced. According to Liu and Hu (2022) [58], in China, the average medical expenses in the pilot areas of long-term care insurance are significantly lower than those in other areas, and the burden of family care is also reduced. Therefore, similar to the systematic long-term care system in developed countries, China needs to build a nationwide long-term care insurance system.

In addition, in cases where family members are unable to provide around-the-clock care for older adults, day and night care facilities, included in long-term care insurance services, can serve as a viable alternative. Alternatively, if family members are the primary caregivers but require ongoing medical services themselves, day and night care services can fulfill the healthcare and nursing needs of the older adults. A study [59] discovered that day and night care services have the capacity to offer tailored nursing services by identifying the specific nursing requirements of older adults and primary caregivers who struggle with ADLs. Day and night nursing services refer the ability of older people with insufficient basic living abilities to receive day or night professional care in a fully equipped nursing institution when family members are unavailable. This nursing service aims to improve the quality of life of older people and maintain their physical and mental health while reducing the physical and mental burden on their families. In the United States, daytime care services have brought home nurses short-term rest care, which has improved the well-being of caregivers and positively impacted the provision of quality services [60]. Therefore, it is necessary to formulate relevant systems that can help improve the quality of care attitude of family caregivers, build nationwide long-term care insurance, and provide professional day and night care facilities.

Based on the results regarding the influence of the main caregivers and their attitude toward the unsatisfactory assistance experience of older adults with disabilities, this study demonstrated the necessity of expanding long-term care-related policies and convalescent institutions. In addition, it is expected to improve the caring attitude of primary caregivers through cost, professional education, and counselling support to maintain intimacy with patients. Therefore, this study can be used as basic data to reduce the lack of assistance for older adults with disabilities.

### Limitations

This study has several limitations. First, it only divided primary caregivers into family and non-family members. In follow-up studies, family members should be further subdivided. Second, the CLHLS data used in this study included only a few factors that may influence the experience of older people with disabilities with unmet assistance. Follow-up studies are necessary if data containing multiple influencing factors emerge. Thirdly, since the ‘unmet aid experience’ in this study is a subjective answer based on personal knowledge, it may be under or over-measured. Fourth, this study is a cross-sectional study using CLHLS data. Fifth, the study includes a significant proportion of participants with no formal education. This is primarily owing to the 8th wave (2018) data consisting of individuals aged 65 and above who were born before the 1950s, a period when illiteracy rates were higher [46]. Such a high proportion can potentially impact the evaluation and interpretation of the relationship between educational level and the outcome variables. Therefore, future research should consider addressing this limitation by examining educational levels in more detail through a finer categorization. Therefore, there are limitations in identifying the causal relationship between the unmet assistance experiences of older adults with disabilities of different gender based on the care attitudes of primary caregivers and caregivers. In follow-up studies, longitudinal data should be used to elucidate the influence of the attitudes of primary caregivers and caregivers on the assistance experience of gender-specific older adults with disabilities.

## Conclusions

In conclusion, the better the caring attitude of caregivers, the less likely older adults with disabilities will experience unsatisfactory assistance. In China, older women with disabilities are more sensitive to main caregivers. However, when the main caregivers are family members, the possibility of experiencing unsatisfactory assistance is low, which is of great significance. Therefore, this study discusses the relevant programs in a targeted manner and considers their reflection on relevant policies and regulations to mitigate the risk of older adults with ADL disabilities having unmet needs for assistance. However, given the limited literature on this topic, out study helps lay a foundation to improve the unmet care experience of the older adults with ADL disabilities depending on gender through follow-up studies. This can contribute to lowering the possibility of unmet care needs for older adults with ADL disabilities.

## Data Availability

The CLHLS datasets are publicly available at the National Archive of Computerized Data on Aging, University of Michigan (http://www.icpsr.umich.edu/icpsrweb/NACDA/studies/36179). Researchers can obtain these data after submitting a data use agreement to the CLHLS team.

## List of abbreviations

ADL: activities of daily living
CLHLS: Chinese Longitudinal Healthy Longevity Survey

## References

[1] Solomons NW (2000). Demographic and nutritional trends among the elderly in developed and developing regions. Eur J Clin Nutr.

[2] Wei L, Yi H, Hai-Lu Z (2019). Elderly people with disabilities in China. J Am Geriatr Soc.

[3] Cieza A, Sabariego C, Bickenbach J, Chatterji S (2018). Rethinking disability. BMC Med.

[4] Uddin MJ, Alam N, Koehlmoos TP, Sarma H, Chowdhury MA, Alam DS (2014). Consequences of Hypertension and Chronic Obstructive Pulmonary Disease, healthcare-seeking behaviors of patients, and responses of the health system: a population-based cross-sectional study in Bangladesh. BMC Public Health.

[5] Wang X, Lin WQ, Chen XJ, Lin Y, Huang L, Zhang SC (2017). Multimorbidity associated with functional independence among community-dwelling older people: a cross-sectional study in Southern China. Health Qual Life Outcomes.

[6] Feng D, Ji L, Xu L (2014). Mediating effect of social support on the association between functional disability and psychological distress in older adults in rural China: does age make a difference?. PLoS ONE.

[7] Barberger-Gateau P, Rainville C, Letenneur L, Dartigues JF (2000). A hierarchical model of domains of disablement in the elderly: a longitudinal approach. Disabil Rehabil.

[8] LaPlante MP, Kaye HS, Kang T, Harrington C (2004). Unmet need for personal assistance services: estimating the shortfall in hours of help and adverse consequences. J Gerontol B Psychol Sci Soc Sci.

[9] DePalma G, Xu H, Covinsky KE, Craig BA, Stallard E, Thomas J, Sands III (2012). Hospital Readmission among older adults who Return Home with Unmet need for ADL disability. Gerontologist.

[10] Desai MM, Lentzner HR, Weeks JD (2001). Unmet need for personal assistance with activities of Daily Living among older adults. Gerontologist.

[11] Hung LC, Liu CC, Kuo HW (2002). Unmet nursing care needs of home-based disabled patients. J Adv Nurs.

[12] LaPlante MP, Kaye HS, Kang T, Harrington C (2004). Unmet need for personal assistance services: estimating the shortfall in hours of help and adverse consequences. The Journals of Gerontology: Series B.

[13] Liu Y-H, Chang H-J, Huang C-C (2012). The unmet activities of Daily Living (ADL) needs of Dependent elders and their related factors: an Approach from both an individual- and area-level perspective. Int J Gerontol.

[14] Otero Á, de Yébenes MJG, Rodríguez-Laso Á, Zunzunegui MV (2003). Unmet home care needs among community-dwelling elderly people in Spain. Aging Clin Exp Res.

[15] Hesketh T (2016). China’s one-child policy—In reply. JAMA.

[16] Tian W, Wu B, Yang Y, Lai Y, Miao W, Zhang X (2022). Degree of protection provided by poverty alleviation policies for the middle-aged and older in China: evaluation of effectiveness of medical insurance system tools and vulnerable target recognition. Health Res Policy Syst.

[17] Chu LW, Chi I (2008). Nursing homes in China. J Am Med Dir Assoc.

[18] Zhu H (2015). Unmet needs in long-term care and their associated factors among the oldest old in China. BMC Geriatr.

[19] Peng R, Wu B, Ling L (2015). Undermet needs for assistance in personal activities of daily living among community-dwelling oldest old in China from 2005 to 2008. Res Aging.

[20] Zunzunegui MV, Alvarado BE, Béland F, Vissandjee B (2009). Explaining health differences between men and women in later life: a cross-city comparison in Latin America and the Caribbean. Soc Sci Med.

[21] Chaoping P, Cen W, Kelifa MO, Xuyang L, Wang P (2023). Gender disparity in disability among Chinese oldest-old: age and cohort trends. J Women Aging.

[22] Botoseneanu A, Allore HG, Mendes de Leon CF, Gahbauer EA, Gill TM (2016). Sex differences in concomitant trajectories of self-reported disability and measured physical capacity in older adults. The Journals of Gerontology: Series A.

[23] Hassan AYI, Lamura G, Hagedoorn M (2022). Predictors of digital support services use by informal caregivers: a cross-sectional comparative survey. BMJ Open.

[24] Sabo K, Chin E (2021). Self-care needs and practices for the older adult caregiver: an integrative review. Geriatr Nurs.

[25] Robinson-Whelen S, Tada Y, MacCallum RC, McGuire L, Kiecolt-Glaser JK (2001). Long-term caregiving: what happens when it ends?. J Abnorm Psychol.

[26] Dionne-Odom JN, Demark-Wahnefried W, Taylor RA, Rocque GB, Azuero A, Acemgil A (2017). The self-care practices of family caregivers of persons with poor prognosis cancer: differences by varying levels of caregiver well-being and preparedness. Support Care Cancer.

[27] Lee M, Kolomer SR (2005). Caregiver burden, Dementia, and elder abuse in South Korea. J Elder Abuse Negl.

[28] Carretero S, Garcés J, Ródenas F, Sanjosé V (2009). The informal caregiver’s burden of dependent people: theory and empirical review. Arch Gerontol Geriatr.

[29] Chen N, Li X, Deng M, Wang CQ, Zhou C (2021). Gender difference in unmet need for assistance with activities of daily living among disabled seniors in China: a cross-sectional study. BMJ Open.

[30] Desin P, Caban-Holt AM, Abner EL, Van Eldik LJ, Schmitt FA. Factors associated with unmet needs among African-American Dementia care providers. J Gerontol Geriatric Res. 2016;5(1). 10.1007/BF03324504. 10.4172/2167-7182.1000267 PMC4864855 27182464

[31] Depalma G, Xu H, Covinsky KE, Craig BA, Stallard E, Thomas JR (2013). Hospital readmission among older adults who return home with unmet need for ADL disability. Gerontologist.

[32] Allen SM, Mor V (1997). The prevalence and consequences of unmet need. Contrasts between older and younger adults with disability. Med Care.

[33] Zhen ZFQG (2015). The impacts of unmet needs for long-term care on mortality among older adults in China. J Disabil Policy Stud.

[34] Andersen RM. Revisiting the behavioral model and access to medical care: does it matter?: J Health Soc Behav 1995:1–10. 7738325

[35] Travers JL, Hirschman KB, Naylor MD (2020). Adapting Andersen’s expanded behavioral model of health services use to include older adults receiving long-term services and supports. BMC Geriatr.

[36] Von Lengerke T, Gohl D, Babitsch B. Re-revisiting the Behavioral Model of Health Care Utilization by Andersen: a review on theoretical advances and perspectives. Health care utilization in Germany: theory, methodology, and results 2013:11–28.

[37] Andersen RM, Davidson PL, Baumeister SE. Improving access to care in America. Changing the US health care system: key issues in health services policy and management 3a edición San Francisco: Jossey-Bass 2007:3–31.

[38] Mohaqeqi Kamal S. H.S.N. Outpatient health service utilization and associated factors among Iranian older adults: based on Andersen’s behavioral model. Educ Gerontol. 2022:1–13.

[39] Hyun SP, Seong AH (2021). Factors influencing unmet healthcare needs among Korean elderly Applied applied by Anderson’s behavioral: gender comparison using Korea Health Panel Survey of 2017. Korean Public Health Research.

[40] Lin W, Yin W, Yuan D (2022). Factors associated with the utilization of community-based health services among older adults in China-an empirical study based on Anderson’s health behavior model. BMC Prim Care.

[41] Heider D, Matschinger H, Müller H, Saum KU, Quinzler R, Haefeli WE (2014). Health care costs in the elderly in Germany: an analysis applying Andersen’s behavioral model of health care utilization. BMC Health Serv Res.

[42] Kim HK, Lee M (2016). Factors associated with health services utilization between the years 2010 and 2012 in Korea: using Andersen’s behavioral model. Osong Public Health Res Perspect.

[43] Willis R, Price D, Glaser K (2013). Ethnicity as a determining factor for instrumental support in mid and later life in England and Wales. J Gerontol B Psychol Sci Soc Sci.

[44] Pan L, Wang C, Cao X, Zhu H, Luo L (2022). Unmet healthcare needs and their determining factors among unwell migrants: a comparative study in Shanghai. Int J Environ Res Public Health.

[45] Zhao Y, Xu X, Dupre ME, Xie Q, Qiu L, Gu D (2020). Individual-level factors attributable to urban-rural disparity in mortality among older adults in China. BMC Public Health.

[46] Zheng Z (2020). Twenty years’ follow-up on elder people’s health and quality of life. China Popul Dev Stud.

[47] Gu D, Feng Q, Zeng Y, Pachana NA (2015). Chinese longitudinal healthy longevity study. Chinese longitudinal healthy longevity study encyclopedia of Geropsychology.

[48] Gu D. General data quality assessment of the CLHLS. Healthy longevity in China: Demographic, socioeconomic, and psychological dimensions 2008:39–60.

[49] Bureau NS (2012). Tabulations of the 2010 China’s Population Census.

[50] He M, Zhou J (2018). The Health differences and influencing factors of elderly in urban and rural areas: based on data of CLHLS 2014. Adv Appl Sociol.

[51] Lee Y-H, Chang Y-C, Chi Y-C, Shelley M. Does urban-rural disparity exist in nicotine and alcohol dependence among Chinese older adults? Addict Res Theory 2023:1–9.

[52] Fu Y, Guo Y, Bai X, Chui EW (2017). Factors associated with older people’s long-term care needs: a case study adopting the expanded version of the Anderson Model in China. BMC Geriatr.

[53] Meng D, Xu G, Davidson PM (2021). Perceived unmet needs for community-based long-term care services among urban older adults: a cross sectional study. Geriatr Nurs.

[54] Zhang Y, Yeager VA, Hou S (2018). The impact of community-based supports and services on quality of life among the elderly in China: a longitudinal study. J Appl Gerontol.

[55] Park SY (2018). The effects of Health-related factors and Social Networks on depressive symptoms in Elderly men and women: focusing on the Moderating effects of gender. Korea Inst Health Social Welf Rev.

[56] Zhong Y, Wang J, Nicholas S (2020). Social support and depressive symptoms among family caregivers of older people with disabilities in four provinces of urban China: the mediating role of caregiver burden. BMC Geriatr.

[57] Shin MH. A study on the expansion plan of home care services for the elderly long-term care insurance in the community care environment. Unpublished master’s thesis, Korea University, Graduate School of Public Administration. 2020.

[58] Liu H, Hu T (2022). Evaluating the long-term care insurance policy from medical expenses and health security equity perspective: evidence from China. Arch Public Health.

[59] Kim NE, Park NH (2022). Activities of Daily Living of the Elderly using Day and Night Care facilities and the nursing needs of the Elderly and their caregivers. Korean Soc Public Health Nurs.

[60] Jeon YH, Brodaty H, Chesterson J (2005). Respite care for caregivers and people with severe mental Illness: literature review. J Adv Nurs.

